# Perspective integration capability: A valid and reliable measurement instrument for assessing knowledge integration readiness in interdisciplinary collaborations

**DOI:** 10.1017/cts.2025.54

**Published:** 2025-04-04

**Authors:** Maritza Salazar Campo, Theresa K. Lant

**Affiliations:** 1 Assistant Professor, Organizations & Management, UCI Paul Merage School of Business, Irvine, CA, USA; 2 Emeritus Distinguished Professor of Management, Pace University, New York, NY, USA

**Keywords:** Interdisciplinary collaboration, knowledge integration, measurement scale, team science, perspective integration

## Abstract

**Background:**

Work in science, medicine, and engineering increasingly relies on collaborations among diverse experts to solve complex problems. Despite the importance of interprofessional training and practice to enhance collaboration and knowledge integration, there is a lack of a conceptually meaningful, valid, and reliable measure of individual capacity for interdisciplinary knowledge integration. This study contributes a conceptual framework and empirical tool to facilitate both research and practice of interdisciplinary collaborations.

**Methods:**

We conduct a three-phase, five-study investigation to develop and validate a measure of individual perspective integration capability (PIC), which assesses individual willingness and ability to integrate knowledge with others during collaborative work. Phase 1 includes item generation and reduction in three studies using different samples of respondents. Phase 2 demonstrates convergent and discriminant validity with conceptually related and unrelated constructs, using a separate sample of respondents. Phase 3 tests criterion-related validity and mediation by examining the multilevel relationships between PIC and key antecedents and outcomes, using data from a unique sample of research scientists in interdisciplinary medical research teams.

**Results:**

Across the three phases of our study, the results demonstrate support for the PIC instrument’s factor structure, reliability, and validity. We also demonstrated that the PIC construct has important implications for individuals engaged in interdisciplinary collaborations.

**Conclusions:**

Having a conceptually meaningful, valid, reliable, and easily administered survey instrument will facilitate further study of interdisciplinary collaboration, and the development and evaluation of integration efforts of teams engaged in convergent and translational initiatives.

## Introduction

Knowledge-based work in domains such as science, medicine, and engineering increasingly relies on collaboration among diverse experts to solve complex problems. Knowledge experts often collaborate in temporary teams on multiple projects simultaneously [1,2]. These collaborations require more than simply sharing expertise; they necessitate deep integration across different knowledge areas [3–5]. Failure to integrate knowledge across specialties can hinder innovation, including the development of diagnostics and treatments for complex diseases. Despite the importance of interprofessional training and practice, collaboration and knowledge integration remain challenging, in large part due to the nature of developing and practicing professional expertise [6,7].

Pursuing professional expertise is a continuous journey of learning and improvement, leading to specialization [8]. Acquiring expertise is rigorous and involves delving into specialized language, thinking, and assessment methods [9]. Experts learn to demonstrate and defend their status and knowledge, serving as role models for novices. Collaborating across professional areas can be hindered by biases that develop along with the development of specialized knowledge [7]. Becoming a disciplinary expert can lead to a fixed mindset, wherein experts view the standards of their profession as superior to different ways of thinking [10]. While specialized expertise is essential, it is also crucial to maintain awareness and openness to knowledge outside one’s area of expertise. Highly trained professionals tend to frame problems in terms of their own expertise. This phenomenon, known as the myopia of learning [11], can create cognitive barriers that make it less likely for one to consider perspectives from different areas of specialization [12].

An understanding of how individual cognitive processing can facilitate or impede knowledge utilization and integration is crucial to the success of interdisciplinary collaborations. Cognitive processing is a critical determinant of knowledge integration among diverse collaborators. Sharing expertise is not sufficient for integration to emerge. Knowledge integration requires deep listening, openness to the contributions of others, thoughtful reflection, and a willingness to update one’s current understanding [13]. It is likely that collaborative knowledge integration begins with a cognitive shift at the individual level, where one contributor recognizes a potential link between their expertise and that of the other. This curiosity about the connection between previously disconnected knowledge unlocks innovative potential, allowing the emergence of new ideas and breakthrough solutions.

Scholars have assumed that the individual-level skill of perspective-taking contributes to forming collective understanding [14]. However, there is still a lack of clarity regarding how considering others’ perspectives [15] leads to a shift in an individual’s cognitive structure. Being receptive to and contemplating the contributions of others is a cognitive capacity that is often overlooked. We refer to this aptitude as a person’s perspective integration capability (PIC). In developing this construct, we drew upon existing theories of collective cognition [16] and cognitive processing [17]. This conceptualization enables an assessment of the degree to which an individual considers inputs from others and how they adjust their current understanding based on the new knowledge received. Building on the conceptual [18,19] and empirical [20,21] literature on interdisciplinary collaboration, this study contributes a conceptual framework and empirical tool to facilitate both research and practice of interdisciplinary collaborations. The instrument we develop assesses an individual’s cognitive openness to the contributions of others and their capacity to integrate new ideas. Conceptually, we set out to develop an instrument that assesses three aspects of an individual’s ability to integrate knowledge with others. First, it measures the extent to which an individual considers the merits of the ideas contributed by collaborators. Once these ideas have been evaluated, the second aspect assesses the individual’s ability to assimilate aspects of those ideas into their task-relevant cognitive schemas. Finally, the third aspect evaluates the individual’s capacity to accommodate aspects of inputs by adjusting task schemas to be more consistent with or complementary to those of collaborators. Our approach addresses the need for practical and reliable measurement tools to assess an individual ability to integrate knowledge with others during interdisciplinary collaborations.

## Materials and methods

We conducted a three-phase, five-study investigation to develop an easily used survey instrument of perspective integration capability (PIC) with desirable psychometric properties. We followed conventional steps for scale development and validation. A large pool of items relevant to knowledge consideration, sharing, elaboration, assimilation, and accommodation, were drawn from the literature on team cognition [16, 17]. The process of scale development and validation typically begins with a large pool of relevant items, which are then reduced and refined using exploratory and confirmatory factor analysis, followed by assessment of convergent and discriminant validity. Details of this process are provided in the Supplement.

Phase 1 includes three studies to develop and validate the PIC scale through exploratory and confirmatory factor analysis. Results show that PIC has two components: Knowledge Consideration (KC) and Knowledge Assimilation/Accommodation (KAA). Although existing literature [16] suggests that knowledge assimilation and knowledge accommodation are two distinct concepts, our results suggest that these concepts overlap significantly. Phase 2 provides evidence for the psychometric properties of PIC by assessing convergent and discriminant validity. In Phase 3, we examine the criterion validity of the PIC instrument. Criterion validity is the last step of a full development and assessment of an instrument’s psychometric properties. It is assessed by analyzing how an instrument behaves in a conceptual model. The PIC instrument developed in this paper focuses on the cognitive aspects of knowledge integration. We use the broader conceptual framework of integrative capacity [19] to explore antecedents and outcomes of PIC using structural equation modeling. The integrative capacity framework suggests how social factors and team emergent states influence knowledge integration in interdisciplinary collaborations. The PIC scale is intended to assess the knowledge integration component of the integrative capacity framework.

### Phase 1: item generation and reduction

In Phase 1, we conducted three studies to develop and validate the PIC scale through exploratory and confirmatory factor analysis. The Phase 1 process involved generating a large list of items, modified from existing measures in the literature on cognitive processes [19], and reduced this number to a feasible set with high content validity [22]. To generate the initial item pool, broad item inclusion criteria were used to circumvent a potential attenuation paradox [23] and used a deductive approach based on existing theory [24]. Details of this process are described in the Supplement. This process yielded 19 items relevant to KC, and 8 items relevant to KAA. All items use 7-point Likert scales with response anchors ranging from “Strongly Disagree” (1) to “Strongly Agree” (7). The pool of initial items and additional details and references describing the methodology are provided in the Supplement.

**Study 1. Exploratory Factor Analysis.** The Study 1 sample was recruited using Amazon’s Mechanical Turk (MTurk). MTurk was selected based on the high diversity of its respondent pool [26], given that participant diversity is highly desirable in the initial phase of scale construction [27]. Respondents were selected for participation if they reported having previously collaborated with team members with diverse, difficult-to-integrate perspectives. They were asked to recall this experience when answering the survey questions. Further data screening filters included answering the entire survey and responding to a neutral language “human intelligence task” that ensured the respondent was not a robot and obscured both the purpose of the research and the respondent qualifications sought by the researchers, thus minimizing self-selection, social desirability, and reactance biases [25]. Study 1 also included items that served as participant screening (4 items), attention checks for data quality (1 item), or were qualitative or demographic items (2 and 5 items, respectively). Two hundred respondents were deemed suitable for inclusion in statistical analyses (53% male, 47% female, mean age 34.6). Iterative exploratory factor analyses (EFA) were conducted to create a cohesive, feasible set of PIC items and to identify factors with demonstrable structural validity and high reliability [28].

Table 1 shows the results of the EFA for Study 1. Principal axis factoring extraction method was used. The rotation method used was Oblimin with Kaiser Normalization. The rotation converged in three iterations. It shows a two-factor solution, with six KC items (*α* = .89) and three KAA items (*α* = .70), for a total of 9 items (*α* = .87). Alpha reliabilities show adequate internal consistency for each factor and the overall PIC scale. Communalities ranged from 0.39 to 0.78, implying that items were adequately associated with their corresponding factors and were correlated, *r* = .61. The KMO (.89) and Barlett’s Test of Sphericity (χ^2^ (36) = 816.15, *p* > .001) suggest that the items can be grouped into underlying factors. Based on the Kaiser rule, the factors explain 54.15% of variance in 9 PIC items.

**Table 1.** Study 1 Exploratory factor analysis pattern matrix

Item	Factor 1	Factor 2
KC1 I carefully evaluate the ideas expressed by each of my team members	0.922	−0.066
KC2 To fully understand the problem, I consider the perspective of each of my team members	0.832	−0.054
KC3 I try to understand the diverse perspectives of my team members	0.791	0.018
KC4 I listen to the viewpoint of each team member, even if it is not widely shared by other members	0.745	−0.033
KC5 Even if my team members have opposing perspectives, I evaluate each in order to consider all issues.	0.660	0.045
KC6 I consider the views of my team members open-mindedly	0.616	0.053
KAA1 My understanding of my work tasks often changes after my team members have shared a different perspective	−0.066	0.744
KAA2 New ideas provided by my team members often change my understanding of how to do something.	0.061	0.624
KAA3 Members of my team share information that causes me to think differently about a work task	0.042	0.596

KC = Knowledge Consideration factor; KAA = Knowledge Accommodation/Assimilation factor.

**Study 2: Exploratory Factor Analysis.** To verify the factor structure of our measure, an EFA was conducted on the 9-item scale resulting from the Study 1 EFA. Undergraduate and graduate students were recruited based on their enrollment in interdisciplinary courses at two private US universities. These courses engaged students in interdisciplinary teamwork by explicitly requiring team composition and outputs to exemplify expertise diversity. Examples of course topics include Environmental Policy Clinic, Crisis Management, e-Learning, and Civic Participation. The nature of the collaborative work assigned to students made them ideal for inclusion in the next step of scale development. The students were participating in interdisciplinary teams at the time that they took the survey, thus providing a closer approximation of actual collaboration than the MTurk sample participants who were asked to recall working in such a team. Student teams were participating in a six-month longitudinal study of interdisciplinary teamwork employing a pretest-posttest design and administration of team training interventions. Data for Study 2 are taken from the pretest stage to prevent any confounds to the structural validity of the measure that might result from differential exposure to the training condition in the study sample. The students were compensated $25.00 per person. The sample included 179 students, 171 of which completed the surveys. Of these respondents, 91 were undergraduate students (63% female, average age 20 years old). Eighty respondents were graduate students (of respondents indicating age and gender, 51% were female, with average age of 33). Additional items included tracking, demographics, and substantive items related to the long-term class project students.

As shown in Table 2, two factors emerged, consistent with Study 1 and verified by parallel analysis in R, explaining 65.30% of the variance in PIC items. Principal axis factoring extraction method was used. The rotation method used was Oblimin with Kaiser Normalization. The rotation converged in four iterations. Data showed high factorability, KMO = 0.91, Barlett’s χ^2^(36) = 971.67, *p* > .001. Communalities ranged from 0.53 to 0.77, and the two factors were correlated, *r* = 0.61, with no meaningful cross-loadings observed between them. Alpha reliabilities show adequate internal consistency (KC *α* = .92; KAA *α* = .86; Composite PIC *α* = .89).

**Table 2.** Study 2 Exploratory factor analysis pattern matrix

Item	Factor 1	Factor 2
KC6 I consider the views of my team members open-mindedly	0.894	−0.031
KC4 I listen to the viewpoint of each team member, even if it is not widely shared by other members	0.874	−0.022
KC3 I try to understand the diverse perspectives of my team members	0.813	−0.064
KC1 I carefully evaluate the ideas expressed by each of my team members	0.777	−0.028
KC5 Even if my team members have opposing perspectives, I evaluate each in order to consider all issues.	0.772	0.023
KC2 To fully understand the problem, I consider the perspective of each of my team members	0.718	0.165
KAA2 New ideas provided by my team members often change my understanding of how to do something	−0.020	0.879
KAA1 My understanding of my work tasks often changes after my team members have shared a different perspective	−0.035	0.761
KAA3 Members of my team share information that causes me to think differently about a work task	0.283	0.523

KC = Knowledge Consideration factor; KAA = Knowledge Accommodation/Assimilation factor.

**Study 3: Confirmatory Factor Analysis.** In a third study, we conducted an analysis to assess the factor structure of the 9-item PIC Index with a confirmatory factor analysis (CFA), using a conservative SEM approach. The respondents were 182 research faculty from five universities participating in a six-month investigation of work interdisciplinary teams. As in Study 2, data used for analysis were collected before any team intervention took place to avoid the potentially confounding effects of receiving different types of training. As incentive, participants were given $250.00 per team to be donated to a charity of their choice. Of the 159 respondents who indicated gender, 44% were male.

Given the challenges of administering surveys to this sample and having an adequate response rate, we had some missing data. Simply deleting the cases with missing information would have made using SEM on the sample inappropriate. Multiple imputation [29] was needed to generate complete responses to satisfy SEM’s high sample size requirements [30]. Analyses were conducted by pooling the results of the SEM model across ten imputed datasets in M*plus* v. 7.3. Confirmatory factor analyses (CFA) were conducted for the measurement model [31,32] and for the PIC structural model. Each measurement model tested revealed good fit (*Knowledge Consideration*: *χ* ^*2*^ = 14.13, *χ* ^*2/*^ */df* = 1.57, *CFI* = 0.98, *TLI* = 0.97, *RMSEA* = 0.06, βs from 0.78 to .90; *Accommodation/Assimilation*: *χ* ^*2*^ = 0.45, *χ* ^*2/*^ */df* = 0.45, *CFI* = 1.00, *TLI* = 1.00, *RMSEA* = 0.00, βs from 0.78 to .86), as did the overall PIC index (*χ* ^*2*^ = 44.41, *χ* ^*2/*^ */df* = 1.71, *p* < 0.05, *CFI* = 0.99, *TLI* = 0.98, *RMSEA* = .06). The high fit indices observed are not unusual for measurement models close to saturation, and equivalence between *χ* ^*2*^ and *χ* ^*2/*^ */df* indicates a single model degree of freedom [33]. No post hoc adjustments were made.

**Summary of Phase 1.** Following recommended procedures for developing valid, reliable, and generalizable survey scales, exploratory and confirmatory factor analyses were conducted in three different samples and results supported a two-factor model to measure PIC. The scale measuring *Knowledge Consideration* consists of six items, and the scale measuring *Knowledge Assimilation/ Accommodation* consists of three items.

### Phase 2: the construct validity of perspective integration capability

To establish a nomological network to validate the PIC measure, we use structural equation modeling to assess convergent and discriminant. We collected a new set of data using MTurk. One hundred twenty-seven respondents were chosen who reported having experience on interdisciplinary teams.

**Study 4: Convergent and Discriminant Validity.** The purpose of Study 4 was to cross-validate the PIC scale with similar constructs in the literature to assess its convergent and discriminant validity. Convergent validity for a focal measure is established when there are significant correlations between the focal measure and measures of similar or dissimilar constructs [34].

Based on prior literature, we chose four conceptually related measures. Each concept was measured with an established scale, which are available in the Supplement. Alpha reliabilities are provided in parentheses. 1) *Bringing Expertise to Bear* (*α* = .88) involves sharing information that is relevant for task completion [35]; 2) *Interpersonal Conflict* (*α* = .72) can reduce willingness to consider another person’s point of view [36]; 3) *Knowledge Hiding* (α > .70) is the intentional attempt of an individual to withhold knowledge [37]; and 4) *Openness to Experience* (*α* = .72) is the extent to which an individual is imaginative, amenable to new ideas and experiences [38]. Hypotheses for the theoretical predictions of the relationship between PIC and the four concepts are listed below.

H1: *Bringing Expertise to Bear* will be positively related to PIC and its components.

H2: *Interpersonal Conflict* will be negatively related to PIC and its components.

H3: *Knowledge Hiding* will be negatively related to PIC and its components.

H4: *Openness to Experience* will be positively related to PIC and its components

Correlational tests of construct validity [39,40] revealed the PIC Index to be positively related to *Bringing Expertise to Bear* and negatively related to *Interpersonal Conflict* and *Knowledge Hiding*. For PIC subscales: *Knowledge Consideration* showed a positive relation with *Bring Expertise to Bear* and *Openness to Experience*, and a negative relationship with *Knowledge Hiding* and *Interpersonal Conflict*. *Knowledge Accommodation/Assimilation* was positively related to *Bringing Expertise to Bear* and negatively related to *Interpersonal Conflict* and *Knowledge Hiding*. Table 3 provides descriptive statistics and correlations. To summarize, study 4 demonstrates convergent and discriminant validity of PIC through its significant but distinct relationships to theoretically derived measures of knowledge processing using Structural Equation Modeling (SEM with MPlus).

**Table 3.** Study 4 Descriptive statistics & correlations of PIC index, PIC factors, & related concepts

Variable	*M*	*SD*	Skew	Kurt.	1	2	3	4	5	6	7
1. Knowledge Consideration	5.74	0.82	−0.82	0.88	1						
2. Knowledge Accommodation /Assimilation	4.90	1.20	−0.33	−0.30	0.45***	1					
3. PIC Index	5.32	0.86	−0.033	−0.29	0.79***	0.91***	1				
4. Bring Expertise to Bear	5.81	0.91	−0.85	0.22	0.56***	0.33***	0.49***	1			
5. Interpersonal Conflict	2.67	0.94	0.34	−0.32	−0.53***	−0.42***	−0.54***	−0.58***	1		
6. Knowledge Hiding	2.70	1.51	0.85	−0.06	−0.42***	−0.31***	−0.31***	−0.43***	0.52***	1	
7. Openness to Experience	4.86	0.70	−0.74	0.80	0.25***	0.03	0.12	0.20*	−0.07	0.07	1

*Note*. *p < 0.05, **p < 0.01, ***p < 0.001. All measures use team as respondent point of reference.

### Phase 3: criterion validity – antecedents and outcomes of PIC

Criterion validity can be assessed by analyzing how a construct behaves in a conceptual model. We tested a model of antecedents and outcomes of PIC using variables proposed in the conceptual model of integrative capacity [19]. The sample we employed for study 5 was 100 members of 26 interdisciplinary teams of scientists. The scientists in this sample all had doctoral training plus several years of postdoctoral education, which emphasized their deep specialization and heightened the barriers to effective coordination of expertise across their specialized areas. Data were collected at two points in time three months apart. The survey at T1 provides data on the antecedent variables in the model as well as the control variable *Interdisciplinary Collaboration Years of Experience.* The survey at T2 provides data on PIC and the outcome variables.

**Figure 1.**
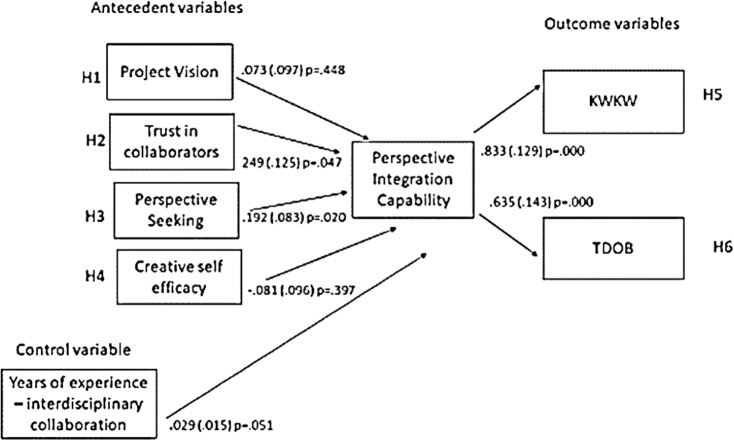
Study 5 Structural equation model.

**Study 5: Structural Equation Modeling.** The conceptual model is illustrated in Figure 1. Drawing from prior research and the model of integrative capacity we identify four antecedents of PIC that foster an orientation to consider, accommodate, or assimilate another’s knowledge. *Project Vision* is a problem conceptualization that characterizes a shared understanding of how unique knowledge of diverse collaborators contributes to the shared task [41]. *Trust* is the trustor’s evaluation of the trustee’s competence, kindness, and honesty, as well as the trustor’s inclination to trust [42]. *Perspective Seeking* refers to actively seeking the perspective of collaborators and indicates a willingness to consider the information received [43,44]. *Creative Self-Efficacy* is an individual’s beliefs about their creative potential and that of collaborators [45]. It motivates participation and is typically a product of a history of member interactions [46].

H1. Project vision is positively related to PIC.

H2. Trust in the competence of collaborators is positively related to PIC.

H3. Perspective-seeking is positively related to PIC.

H4. Creative self-efficacy is positively related to PIC.

Consistent with the integrative capacity model [19], we also examine two important outcomes that PIC will likely enhance. Knowing who knows what (KWKW), a subcomponent of transactive memory, refers to being aware of what your collaborators know [47]. Higher levels of PIC are expected to enhance the understanding of where expertise is located among collaborators. *Transdisciplinary orientation behavior (TDOB)* measures a willingness to consider ideas from collaborators outside your own area of expertise by engaging in specific behaviors to learn more about their fields [21]. As PIC increases, individuals will become more open to the insights, tools, and approaches possessed by their collaborators.

H5: PIC enhances the knowledge of where expertise resides among collaborators (KWKW).

H6. PIC enhances the willingness to draw on knowledge, methods, and approaches from other disciplines (TDOB).

Lastly, we hypothesize that PIC mediates the relationships between the proposed sets of antecedents and outcomes.

H7. PIC mediates the relationship between the theoretically relevant antecedents (vision, trust, perspective seeking, and creative self-efficacy) and outcomes (KWKW and TDOB).

Table 4 provides descriptive statistics and a correlation matrix. To test the relationship between the antecedents, PIC, and outcomes, we used maximum likelihood estimation in MPlus version 8.3 [48] to consider the longitudinal nature of the data. The analysis made use of 2,000 bootstrapped samples of indirect (a*b), direct (path c’), and total effects [49]. The model fit the data well (*χ* ^*2*^/df = 1.82, CFI = 0.91, RMSEA = .09).

**Table 4.** Study 5. Descriptive statistics & correlations of PIC index with antecedents and outcomes

Variable	*M*	SD	Skew	Kurt	1	2	3	4	5	6	7
1. PIC Index	6.08	0.57	−0.54	1.16							
2. Knowing Who Knows What	6.30	0.76	−1.37	3.06	0.63						
3. Transdisciplinary Orientation Behavior	5.98	0.81	−0.57	−0.47	0.45	0.39					
4. Years of Experience	4.34	3.65	0.45	−1.22	0.15	−0.01	0.06				
5. Trust in Collaborators	6.33	0.67	−1.21	1.81	0.36	0.35	0.16	−0.08			
6. Creative Self-Efficacy	5.68	0.96	−0.32	−0.09	0.25	0.33	0.21	−0.04	0.49**		
7. Perspective Seeking	5.55	0.93	−0.53	0.01	0.39	0.37	0.30	−0.05	0.31**	0.54**	
8. Project Vision	5.76	1.00	−0.72	−0.01	0.32	0.43	0.21	0.01	0.41**	0.67**	0.52**

*Note*. ** *p* < .001.

As shown in Figure 1, the control variable *years of experience* is positively related to PIC. Hypotheses 2 and 3 are supported, as antecedents *trust* and *perspective seeking* are positively related to PIC. Hypotheses 5 and 6 are supported, with outcomes KWKW and TDOB being positively associated with PIC. Hypothesis 7 predicts a mediation relationship between antecedents and outcomes through PIC. The strength and direction of the path from the antecedent to the mediator and from the mediator to the outcome are used to assess this indirect effect. For the relationships between the antecedent and outcome variables as mediated by PIC, the indirect effects were significant for *perspective-seeking* to TDOB (*B* = 0.14, *p* < .05; .95% CI [−0.02, .30]), and marginally significant for *trust* to KWKW (*B* = 0.18, *p* < .10; 95% CI [−0.01, .43])

## Discussion and conclusion

The set of studies in this manuscript aimed to create a reliable and valid measure of the perspective integration capability of individuals working in expertise diverse collaborations. We conducted a three-phase, five-study investigation to develop and validate a measure of individual perspective integration capability (PIC), which assesses individual ability to integrate knowledge with others during collaborative work. The results demonstrate the scale’s validity, reliability, and generalizability across differing populations and showed that PIC is related to but distinct from other constructs.

In Phase 1, the results of the exploratory and confirmatory factor analysis in three studies using different samples of respondents yielded a nine-item, two-factor measurement scale. These two factors are *Knowledge Consideration* and *Knowledge Accommodation/Assimilation*. Phase 2 established convergent and discriminant validity using conceptually related and unrelated constructs. The results of structural equation modeling using data from an independent sample of respondents support the two-factor PIC measure. Our results indicated that PIC was positively related to *bringing knowledge to bear,* whereas it had a negative association with *knowledge hiding* and *interpersonal conflict*. Phase 3 tested criterion-related validity and mediation by examining the multilevel relationships between PIC and theoretically relevant antecedents and outcomes. Using data from a unique sample of research scientists in interdisciplinary medical research teams at two points in time, structural equation modeling established criterion and predictive validity of the scale. This analysis provides further insight into how the perspective integration construct can add to our understanding of individual cognitive capability to utilize and integrate diverse perspectives in collaborative work. Our analysis revealed support for two antecedents, *perspective seeking* and *trust*, and two outcomes, namely *knowing who knows what* and t*ransdisciplinary orientation* behaviors.

This manuscript makes several contributions to research and practice concerning diverse collaborations that require knowledge integration. Such collaborations require more than simply sharing expertise; they necessitate deep integration across different knowledge areas. Despite the importance of interprofessional training and practice, collaboration and knowledge integration remain challenging. Collaborating on knowledge-intensive tasks requires the ability to evaluate and utilize knowledge effectively [13,16]. The conceptual framework of integrative capacity in diverse collaborations [19] suggests that perspective integration is essential for navigating the complexities of diverse knowledge collaboration. The initial step in joint knowledge creation involves assessing and sharing each other’s knowledge through knowledge consideration. Cognitive integration entails the incorporation of new knowledge into existing frameworks. Assimilation occurs when individuals encounter information that aligns with their current understanding, whereas accommodation occurs when individuals encounter information that challenges or contradicts their existing beliefs or knowledge. Knowledge integration through assimilation, therefore, consists of the incorporation of novel information into current understanding and accommodation requires adjusting mental schemas to incorporate novel information. This dynamic between assimilation and accommodation is essential to the active and evolving nature of cognition. It highlights how individuals continually shape and refine their mental models in response to the ever-expanding landscape of learning and experience.

We suggest that one barrier to successful interdisciplinary collaboration is the lack of a psychometrically valid and easily administered measurement instrument to provide data to collaborators, team leaders, and research development professionals about a team’s readiness to integrate their diverse expertise. Across the three phases of our study, the results demonstrate support for the PIC instrument’s factor structure, reliability, and validity. We also demonstrate that the construct of PIC has important implications for individuals engaged in interdisciplinary collaboration. Having a conceptually meaningful, valid, reliable, and easily administered survey instrument will facilitate further study of interdisciplinary collaboration and the development and evaluation of integration efforts of teams engaged in convergent and translational initiatives. This set of studies demonstrates that the PIC measurement instrument is a valid, reliable, and conceptually meaningful for studying collaborations.

Finding solutions to important problems like identifying cures for complex disease or enhancing the effectiveness of robotics in manufacturing, requires drawing expertise from disparate fields. In these problem-focused teams, experts, each with their unique and specialized knowledge, are brought together and charged with the difficult task of working together to arrive at a joint solution. This requires the integration of their knowledge and engaging in processes assessed in the PIC scale including knowledge consideration and knowledge assimilation/accommodation. The PIC scale can be leveraged as a diagnostic tool to assess the extent to which knowledge integration is occurring within interdisciplinary and translational teams. Assessing knowledge integration over time has become an important metric to government funding agencies. Having a tool to rapidly and accurately assess team member readiness for cognitive integration can be a competitive advantage for teams seeking federal funding. Moreover, our final study demonstrates how the scale can be used to assess change in knowledge integration capability over time, which can be useful to track team development.

In summary, our aim was to develop and validate a measure of individual-member perspective integration capability, which can assess individuals’ willingness and ability to integrate knowledge with others as part of their work. This scale focuses on evaluating whether an individual is open to the contributions of others and whether they can adopt new ideas or insights they receive. We provide a reliable scale to measure one’s ability to integrate knowledge, which we tested for validity in different contexts and samples. Our results demonstrated the scale’s generalizability across populations and show that PIC is related to but distinct from other constructs. Third, we are the first to empirically show the importance of PIC in collaborative and cross-boundary work environments. PIC can predict individuals’ knowledge of who knows what and their willingness to draw upon and use expertise from other disciplines.

### Strengths, limitations, and future directions

The strength of this set of studies is the application of well-established standards in creating valid and reliable survey instruments to team science, interdisciplinary and translational research. The scale developed fills a critical need in both research and practice to have a psychometrically established instrument that is easy to use. The limitations of the study are that the aggregate traits of the samples or our choice of outcome variables may have biased the results. For example, all individuals across the five studies shared a characteristic of having interdisciplinary education or work experience. The omission of respondents without this characteristic, while important to sample selection, eliminates the possibility of identifying boundary conditions on the application of the PIC instrument. The PIC scale items are self-reports, which can lead to bias based on the Dunning-Kruger effect [50] where less skilled individuals tend to overestimate their ability more than highly skilled individuals. Future research is needed to further explore the validity and applicability of the PIC scale. Although it is challenging to study individuals and teams in the field, we encourage more research on interdisciplinary and translational teams in action.

A practical implication of our work is that information gathered using this tool can be used to assess the initial conditions of a collaborative team, to guide professional development activities, and to assess the growth of a team’s integrative capacity over time. Organizations could develop training to increase perspective integration capacity. Efforts to build trust among collaborators and willingness to seek others’ perspectives should increase PIC, which, in turn, could increase the likelihood of knowledge integration through an increase in transdisciplinary orientation behaviors and knowledge of who knows what in a collaboration. Moreover, leadership training programs that develop leaders who promote and reinforce behaviors that foster a perspective integrative capability will be impactful for teams and organizations who want to improve utilization and integration of available knowledge.

## Conclusion

Our research on perspective integration offers a solid foundation for building upon prior theoretical and empirical work and creates new avenues for future investigation. Perspective integration is a process that occurs at the individual level and influences how knowledge is shared between individuals and larger groups such as teams or departments, which has excellent potential for future research. We anticipate studies that build on the conceptual framework developed in this paper and empirically validated measurement for perspective integration. We also expect the easily administered questionnaire instrument will be used in practice to enhance interdisciplinary collaboration.

## Supporting information

Salazar Campo and Lant supplementary material Salazar Campo and Lant supplementary material

## Supplementary material

The supplementary material for this article can be found at https://doi.org/10.1017/cts.2025.54.

## References

[1] Mistry S , Kirkman BL , Moore O , et al. Too many teams? Examining the impact of multiple team memberships and permanent team identification on employees’ identity strain, cognitive depletion, and turnover. Pers Psychol. 2023;76(3):885–912. doi: 10.1111/peps.12515.

[2] O’Leary MB , Mortensen M , Wooly AW. Multiple team membership: a theoretical model of its effects on productivity and learning for individuals and teams. Acad Manage Rev. 2011;36(3):461–478. doi: 10.5465/amr.2009.0275.

[3] Sanders, S. National Academies of Sciences, Engineering, and Medicine. Measuring convergence in science and engineering: Proceedings of a workshop. The National Academies Press, 2021. doi: 10.17226/26040.

[4] The National Academies Keck Futures Initiative. National Research Council. Collaborations of consequence: NAKFI’s 15 years igniting innovation at the intersections of disciplines. The National Academies Press, 2018. doi: 10.17226/25239. 30640415

[5] Petersen AM , Ahmed ME , Pavlidis I. Grand challenges and emergent modes of convergence science. Humanit Soc Sci Commun. 2021;8(194):1–15. doi: 10.1057/s41599-021-00869-9.38617731

[6] Kumarasamy MA , Sanfillippo FP. Breaking down silos: engaging students to help fix the US healthcare system. J Multi Health. 2015;8:101–108. doi: 10.2147/JMDH.S79384. PMC4337621 25733912

[7] Rider EA , Chou C , Abraham C , et al. Longitudinal faculty development to improve interprofessional collaboration and practice: a multisite qualitative study at five US academic health centers. BMJ Open. 2023;13(4):e069466. https://pubmed.ncbi.nlm.nih.gov/37076167/. 10.1136/bmjopen-2022-069466 PMC10124268 37076167

[8] Snell R , Fyfe SF , et al. Development of professional identity and professional socialization in allied health students: a scoping review. Focus Health Prof Edu. 2020;21(1):29–56.

[9] Hall K , Vogel A , Stipelman B , et al. A four-phase model of transdisciplinary team-based research: goals, team processes and strategies. Trans Behav Med. 2012;2(4):415–430. 10.1007/s13142-012-0167-y PMC3589144 23483588

[10] Lombardo L , Ehlers J , Lutz G. Mindset and reflection - how to sustainably improve intra-and interpersonal competencies in medical education. Healthcare. 2023;11(6):859. doi: 10.3390/healthcare11060859. 36981516 PMC10048539

[11] Levinthal DA , March JG. The myopia of learning. Strategic Manage J. 1993;14(52):95–112. doi: 10.1002/smj.4250141009.

[12] Clark KM. Interprofessional education: making our way out of the silos. Resp Care. 2018;63(5):637–639. doi: 10.4187/respcare.06234. 29703797

[13] Harvey S. A different perspective: the multiple effects of deep level diversity on group creativity. J Exp Soc Psychol. 2013;49(5):822–832. doi: 10.1016/j.jesp.2013.04.004.

[14] Hoever IJ , van Knippenberg D , van Ginkel WP , et al. Fostering team creativity: perspective taking as key to unlocking diversity’s potential. J Appl Psychol. 2012;97(5):982–996. doi: 10.1037/a0029159. 22774764

[15] Parker SK , Axtell CM. Seeing another viewpoint: antecedents and outcomes of employee perspective taking. Acad Manag J. 2001;44(6):1085–1100. https://www.jstor.org/stable/3069390.

[16] Gibson CB. From knowledge accumulation to accommodation: cycles of collective cognition in work groups. J Organ Behav. 2001;22(2):121–134. https://www.jstor.org/stable/3649586.

[17] Homan AC , van Knippenberg D , Van Kleef GA , et al. Interacting dimensions of diversity: cross-categorization and the functioning of diverse work groups. Group Dyn - Theor Res. 2007;11(2):79–94. doi: 10.1037/1089-2699.11.2.79.

[18] Falk-Krzesinski H , Borner K , Contractor N , et al. Advancing the science of team sciences. Clin Transl Sci. 2010;3(5):263–266. 20973925 10.1111/j.1752-8062.2010.00223.x PMC2965626

[19] Salazar MR , Lant TK , Fiore SM , et al. Facilitating innovation in diverse science teams through integrative capacity. Small Gr Res. 2012;43(5):527–558. doi: 10.1177/1046496412453622.

[20] Lotrecchiano GR , Schwartz L , Falk-Krzesinski HJ. Measuring motivation for team science collaboration in health teams. Clin Transl Sci. 2020;5(e84):1–6. doi: 10.1017/cts.2020.567. PMC8111607 34007467

[21] Misra S , Stokols D , Cheng L. The transdisciplinary orientation scale: factor structure and relation to the integrative quality and scope of scientific publications. J Trans Med Epid. 2015;3(2):1042.

[22] Floyd FJ , Widaman KF. Factor analysis in the development and refinement of clinical assessment instruments. Psych Assess. 1995;7(3):286–299. doi: 10.1037/1040-3590.7.3.286.

[23] Loevinger J. The attenuation paradox in test theory. Psych Bul. 1954;51(5):493–504. doi: 10.1037/h0058543. 13204488

[24] Hinkin TR. A brief tutorial on the development of measures for use in survey questionnaires. Organ Res Meth. 1998;1(1):104–121. doi: 10.1177/109442819800100106.

[25] Schulze T , Krug S , Schader M. Workers’ task choice in crowdsourcing and human computation markets. In: ICIS. 2012. Proceedings: 33rd International Conference on Information Systems

[26] Buhrmester M , Kwang T , Gosling SD. Amazon’s mechanical turk: a new source of inexpensive, yet high-quality, data? Perspect Psych Sci. 2011;6(1):3–5. doi: 10.1177/1745691610393980. 26162106

[27] Clark LA , Watson D. Constructing validity: basic issues in objective scale development. Psych Assess. 1995;7(3):309–319. doi: 10.1037/1040-3590.7.3.309.

[28] Comrey AL. Factor-analytic methods of scale development in personality and clinical psychology. J Consult Clin Psych. 1988;56(5):754–761. doi: 10.1037/0022-006X.56.5.754. 3057010

[29] Rubin DB. Multiple imputation after 18+ years. J Am Stat Assoc. 1996;91(434):473–489. https://www.jstor.org/stable/pdf/2291635.pdf.

[30] Wolf EJ , Harrington KM , Clark SL , et al. Sample size requirements for structural equation models: an evaluation of power, bias, and solution propriety. Educ Psych Measur. 2013;73(6):913–934. doi: 10.1177/0013164413495237. PMC4334479 25705052

[31] Hu L , Bentler PM. Evaluating model fit. In: Hoyle RH , ed. Structural equation modeling: Concepts, issues, and applications. Sage Publications, Inc, 1995: 76–99.

[32] Hu L , Bentler PM. Cutoff criteria for fit indexes in covariance structure analysis: conventional criteria versus new alternatives. Struct Equat Model. 1999;6(1):1–55. doi: 10.1080/10705519909540118.

[33] Mulaik SA , James LR , Van Alstine J , Bennett N , Lind S , Stilwell CD. Evaluation of goodness-of-fit indices for structural equation models. Psych Bul. 1989;105(3):430–445. doi: 10.1037/0033-2909.105.3.430.

[34] Schwab DP. Research methods for organizational studies. Psychology Press, 2013.

[35] Faraj S , Sproull L. Coordinating expertise in software development teams. Manage Sci. 2000;46(12):1554–1568. http://www.jstor.org/stable/2661533.

[36] Knight DP , Smith CL , et al. Top management team diversity, group process, and strategic consensus. Strat Manag J. 1999;20:445–465.

[37] Connelly CE , Zweig D , Webster J , et al. Knowledge hiding in organizations. J Organ Beh. 2012;33(1):64–88. https://www.jstor.org/stable/pdf/41415737.pdf.

[38] Hislop D. Knowledge integration processes and the appropriation of innovations. Europ J Innov Manag. 2003;6(3):159–172. doi: 10.1108/14601060310486235.

[39] Morizot J. Construct validity of adolescents’ self-reported big five personality traits: importance of conceptual breadth and initial validation of a short measure. Assess. 2014;21(5):580–606. doi: 10.1177/1073191114524015. 24619971

[40] Cronbach L , Meehl PE. Construct validity in psychological tests. Psych Bul. 1955;52(4):281–302. doi: 10.1037/h0040957. 13245896

[41] van Ginkel WP , van Knippenberg D. Group leadership and shared task representations in decision making groups. Leader Quar. 2012;23(1):94–106. doi: 10.1016/j.leaqua.2011.11.008.

[42] Mayer RC , Davis JH , Schoorman FD. An integrative model of organizational trust. Acad Manag Rev. 1995;20(3):709–734. doi: 10.2307/258792.

[43] Davis MH , Conklin L , Smith A , et al. Effect of perspective taking on the cognitive representation of persons: a merging of self and other. J Pers Soc Psych. 1996;70(4):713–726. doi: 10.1037/0022-3514.70.4.713. 8636894

[44] Grant AM , Berry J. The necessity of others is the mother of invention: intrinsic and prosocial motivations, perspective taking, and creativity. Acad Manag J. 2003;54(1):73–96. doi: 10.5465/AMJ.2011.59215085.

[45] Shin SJ , Zhou J. When is educational specialization heterogeneity related to creativity in research and development teams? Transformational leadership as a moderator. J Appl Psych. 2007;92(6):1709–1721. doi: 10.1037/0021-9010.92.6.1709. 18020807

[46] Ford CM. A theory of individual creative action in multiple social domains. Acad Manag Rev. 1996;21:1112–1142. doi: 10.2307/259166.

[47] Lewis K. Measuring transactive memory systems in the field: scale development and validation. J Appl Psych. 2003;88(4):587–604. 10.1037/0021-9010.88.4.587 12940401

[48] Muthén LK , Muthén BO. Mplus User’s Guide, 8th Edition ed. 1998-2017. https://www.statmodel.com/download/usersguide/MplusUserGuideVer_8.pdf.

[49] Baron RM , Kenny DA. The moderator-mediator variable distinction in social psychological research: conceptual, strategic, and statistical considerations. J Pers Soc Psych. 1986;51(6):1173–1182. doi: 10.1037//0022-3514.51.6.1173. 3806354

[50] Dunning D. The dunning-kruger effect: on being ignorant of one’s own ignorance. In: eds. Advances in experimental social psychology. Academic Press, 2011;44:247–296.

