# Supplementary material for: Perspective integration capability: A valid and reliable measurement instrument for assessing knowledge integration readiness in interdisciplinary collaborations
Source: J Clin Transl Sci. 2025 Apr 4;9(1):e93. doi: 10.1017/cts.2025.54 (PMC12089850; doi:10.1017/cts.2025.54)
Supplement: Salazar Campo and Lant supplementary material [file S2059866125000548sup001.docx]

**Supplemental Information**

The initial item generation process drew from prior field work conducted by the authors in a large medical institution. This field work involved over 100 hours of observation and interviews with interdisciplinary research team participants. The integrative capacity (IC) framework [1] was developed based on this field work. This framework was highlighted in the NASEM publication on facilitating team science [2]. Measures of leader behaviors, team social integration behaviors and emergent states from the IC framework have been developed and explored in other publications [3, 4]. In the IC model, these factors are antecedents to cognitive integration. The set of studies in this manuscript focuses exclusively on measuring the cognitive aspects of knowledge integration. To develop the measure, we followed conventional steps for scale development and validation [5].

The table below summarizes the three phases and five studies.

| **Phase/Study** | | **Sample Source** | **Sample Description & Demographics** | **Measures** | **Analysis** | **Key Findings** |
| --- | --- | --- | --- | --- | --- | --- |
| **Phase 1**  **Item Generation & Reduction** | **Study 1**:  Scale development | MTURK | 200 professionals with interdisciplinary team experience.  53% Male, 47% female, Mean age 35. | Pool of 27 PIC items, plus attention checks, screening items, demographics | Exploratory Factor Analysis  IBM SPSS 22  PAF extraction | 2-factor solution; reduced set of items. Knowledge Consideration, α = .89.  Accommodation/ Assimilation, α = .70.  Composite PIC, α = .87. |
|  | **Study 2**: Scale validation / replication | University graduate & UG students | 91 UG and 80 grad students conducting interdisciplinary projects. | Reduced set of items - 9 PIC items | Exploratory Factor Analysis  IBM SPSS 22 / R | Knowledge Consideration, α = .92.  Accommodation/Assimilation, α = .86.  Composite PIC, α = .89. |
|  | **Study 3:** Factor structure replication and validation | University research faculty | 182 research faculty (5 universities) in interdisciplinary teams  44% Male, 56% Female; 13 didn't say. | 9 PIC items | Confirmatory Factor Analysis  MPlus structural equation modeling | Knowledge Consideration, α = .94.  Accommodation/Assimilation, α = .86.  Composite PIC, α = .92. |
| **Phase 2 - Convergent & Discriminant Validity** | **Study 4**:  Assess convergent and discriminant validity | MTURK | 127 professionals with interdisciplinary team experience  52 % male, 48% female; 77% Mean Age: 38, | 9 PIC items and 22 items for 4 related concepts | MPlus structural equation modeling | PIC positively related to Bring Expertise to Bear, *r* = .49, *p* < .001,  PIC inversely related to Knowledge Hiding *r* = -.31, *p* < .001 and Interpersonal Conflict r=-54, *p*<.001 |
| **Phase 3 – Criterion / Predictive Validity** | **Study 5**:  Assess criterion validity in model with antecedents & outcomes | University research faculty | 100 research faculty working in interdisciplinary teams  35% female  59% male  Remainder other /missing | 9 PIC items and 18 items for 4 antecedents and 9 items for 2 outcome variables. | MPlus structural equation modeling | Antecedents of PIC: Trust *b*=.249 *p*=.047 and Perspective Taking *b*=.192, *p*=.020. Outcomes of PIC: KWKW *b*=.833, p=.000 and TDOB *b*=.635, *p*=.000 |

Note: *a =* Cronbach alpha reliabilities; *r* = correlation coefficient; *b* = estimation coefficient

**Preliminary Scale Development: Item Generation and Item Selection**

The initial large pool of potential survey items was generated from existing studies of team collaboration and cognition. The authors searched a wide variety of studies that examined team collaboration processes in a number of contexts, and organized these potential items based on their relevance to knowledge consideration, knowledge assimilation, and knowledge accommodation. This process yielded the initial pool of items described below.

**Phase 1 Item Generation & Reduction**

The PIC item generation process yielded nineteen items relevant to knowledge consideration, and eight items relevant to knowledge assimilation/accommodation. Items were created and presented in a manner consistent with best practice empirical principles of survey design to maximize respondent comprehension, engagement, and the likelihood of receiving a genuine, thoughtful response. All items used 7-point Likert scales ranging from “Strongly Disagree” (1) to “Strongly Agree” (7).

**Study 1.**

The full pool of 27 items is listed below. For each construct, the items that were maintained in the EFA process are listed first, in bold. The items that were removed are listed second, in italics. The sample for study 1 is 200 MTurk respondents. MTurk respondents tend to be somewhat younger than the general working population, although gender is a roughly comparable [6]. Two hundred and sixteen respondents were recruited for study 1. Although some have recommended much larger sample sizes for the initial stage of scale construction the number of respondents obtained (N > 200) adequately reflects a sample size capable of feasibly promoting a “heterogeneity of irrelevancies” among participant responses, and facilitates ultimate generalizability of the results to the populations represented [7-9]

The EFAs were performed in IBM SPSS 22. All EFAs used principal axis factoring (PAF) extraction, ideal for determining underlying dimensions [10]. Each EFA also used direct oblimin rotation, an oblique rotation permitting correlated factors [11]. The factors were allowed to emerge instead of specifying a number in advance to bolster confidence in results. Item deletion criteria followed best practice recommendations, including screening for low factor loading or high relative cross loading, poor wording or conceptual redundancy, and low operational specificity [12].

**Knowledge Consideration**

1. **I carefully evaluate the ideas expressed by each of my team members.**
2. **To fully understand the problem, I consider the perspective of each of my team members.**
3. **I try to understand the diverse perspectives of my team members.**
4. **I listen to the viewpoint of each team member, even if it is not widely shared by other members.**
5. **Even if my team members have opposing perspectives, I evaluate each in order to consider all the issues.**
6. **I consider the views of my team members open-mindedly.**
7. *Before seeking a solution, I carefully consider the views of my team members in order to understand the problem.*
8. *I actively try to consider the ideas of my team members.*
9. *I look to my team members for different interpretations of how to confront a problem.*
10. *I often consider the different opinions of my team members for the sake of obtaining optimal outcomes for the team.*
11. *I try to take all possible perspectives into consideration.*
12. *I seek to understand what my team members are trying to do.*
13. *I enjoy comparing the different views of my team members.*
14. *We all consult the perspectives of other team members before making decisions on a project.*
15. *In risky situations, the discussions that we have in this team reflect multiple points of view.*
16. *During conversations, I listen carefully to my team members in order to obtain as much information as possible.*
17. *I criticize the suggestions of my team members in order to better understand their perspectives.*
18. *I freely challenge the assumptions made by my team members when I am trying to evaluate their ideas.*
19. *I judge the weak points in my team members’ perspectives in order to figure out how valuable their ideas are.*

**Knowledge Accommodation/Assimilation**

1. **My understanding of my work tasks often changes after my team members have shared a different perspective.**
2. **New ideas provided by my team members often change my understanding of how to do something.**
3. **Members of my team share information that causes me to think differently about a work task**
4. *The knowledge that my team members contribute makes it easy for me to connect my perspective to theirs.*
5. *It is easy for me to see how the knowledge held by my team members is compatible with my own.*
6. *I find it easy to combine ideas suggested by my team members with my own understanding of how to approach my work.*
7. *It is easy for me to adapt my ideas to fit with the suggestions of my team members.*
8. *I try to incorporate the views of my team members into my own perspective.*

**Study 2**: The nine-items remaining after Study 1 EFA are used in Study 2 for scale validation and replication using EFA in a separate sample of respondents. These nine items are listed below in the order in which they loaded into factors in Study 1.

**Knowledge Consideration**

1. I carefully evaluate the ideas expressed by each of my team members.
2. To fully understand the problem, I consider the perspective of each of my team members.
3. I try to understand the diverse perspectives of my team members.
4. I listen to the viewpoint of each team member, even if it is not widely shared by other members.
5. Even if my team members have opposing perspectives, I evaluate each in order to consider all the issues.
6. I consider the views of my team members open-mindedly.

**Knowledge Accommodation/Assimilation**

1. My understanding of my work tasks often changes after my team members have shared a different perspective.
2. New ideas provided by my team members often change my understanding of how to do something.
3. Members of my team share information that causes me to think differently about a work task

**Study 3:** The nine item factor structure was replicated in Study 2. The same nine items are included in the Study 3 factor structure replication using confirmatory factor analysis in a separate sample of respondents.

**Study 4:** The nine item PIC instrument was analyzed for convergent and discriminant validity by examining its relationship to a number of conceptually related concepts. The concepts and their associated scales are provided below.

**Bringing Expertise to Bear**: Adapted from the Bring Expertise to Bear [13]

1. I share my expertise with other team members by making suggestions.
2. I make suggestions to help my team reach its goals.
3. When I have more expertise to perform a difficult task, I share my knowledge and skills with my team members.

**Interpersonal conflict*.*** Adapted from [14].

1. Relationships between members of the team are best described as “win-lose,” if he/she wins, I lose
2. Relationships between members of the team are positive and rewarding (reverse coded)
3. Team members are always ready to cooperate and help each other. (reverse coded)
4. Team members really stick together (reverse coded)
5. Team members get along well together. (reverse coded)

***Knowledge hiding.*** Adapted from evasive hiding subscale [15]

1. In general, the members of this team agree to help each other but never really intend to
2. In general, the members of this team offer each other some other information instead of what they really wanted
3. In general, the members of this team say they will help each other but stall as much as possible.
4. In general, the members of this team agree to help each other but do not provide the information requested

**Openness to experience*.*** Adapted from [16]

1. I see my team as a group of individuals who are curious about many different things
2. I see my team as a group of individuals who are original, often have new ideas.
3. I see my team as a group of individuals who are ingenious and reflect a lot
4. I see my team as a group of individuals who have a lot of imagination
5. I see my team as a group of individuals who are inventive and creative
6. I see my team as a group of individuals who like artistic or aesthetic experiences
7. I see my team as a group of individuals who are not really interested in different cultures, their customs, and valued (reverse coded).
8. I see my team as a group of individuals who like to reflect and try to understand complex things
9. I see my team as a group of individuals who have few artistic interests (reverse coded)
10. I see my team as a group of individuals who are sophisticated when it comes to art, music or literature

**Study 5:** The nine item PIC instrument was analyzed for criterion validity by analyzing its relationship to antecedents and outcomes from a broader conceptual model. The scales for the antecedents and outcomes are provided below.

**Antecedents:**

**Project vision** Adapted from [17-18]

The team leader...

1. Articulates a compelling vision of the future for our team.
2. Talks enthusiastically about what needs to be accomplished by our team.
3. Communicates a clear direction of where our team is going.
4. Provides a clear vision of where our team is going.
5. Because of my team leader, I have a clear vision of our team’s purpose.

**Trust in competence of collaborators** Adapted from [19]

1. I feel very confident about the skills of the other department.

2. The members of the other department have much knowledge about the work that needs to be done.

3. The members of the other department have specialized capabilities that can increase our performance.

4. The members of the other department are well qualified.

5. The members of the other department are very capable of performing their tasks.

6. The members of the other department seem to be successful in the activities they undertake.

**Perspective seeking**

1. I ask questions in an effort to see the world through my team members’ eyes.

2. I make inquiries to understanding my team members were feeling.

3. I inquire about my team members’ viewpoints to understand them better.

4. I ask questions to learn about my team members’ perspectives.

**Creative self-efficacy** Adapted from [20]

1. I have confidence in my team’s ability to solve problems creatively.

2. The members in my team have a knack for developing the ideas of others.

3. My team is good at generating novel ideas

**Outcomes**:

**Knowing who knows what** adapted from [21]

1. If I need to get expertise on a certain issue, I know exactly who to turn to in this team.

2. I know which team members have expertise in certain areas.

3. I have a good understanding of “who knows what” in this team.

**Transdisciplinary orientation behavior**

1. I generally approach problems from a multi-level perspective that encompasses both micro and

macro-level factors.

2. My research projects to date reflect my ability to conceptualize complex problems by

identifying various situation-specific factors that account for those problems.

3. My research to date reflects my ability to create conceptual frameworks that bridge multiple

fields.

4. My research projects to date reflect my ability to think broadly about complex problems.

5. In my own work, I incorporate perspectives from fields that are different from my own.

6. In my own research, I use research methods drawn from more than one discipline rather than relying exclusively on a single disciplinary approach.

**Supplement References**

1. Salazar M. Lant T. Facilitating Innovation in Interdisciplinary Teams: The Role of Leaders and Integrative Communication, *Informing Science: The International Journal of an Emerging Transdiscipline, 2018 21, 157-178,* <https://doi.org/10.28945/4011>
2. National Research Council. *Enhancing the Effectiveness of Team Science*. Washington, DC: The National Academies Press. 2015 https://doi.org/10.17226/19007
3. Salazar M Doiron K Widmer K Lant T. Leader Integrative Capabilities: A Catalyst for Effective Inter-disciplinary Teams in *Advancing Social and Behavioral Health Research through Cross-Disciplinary Team Science: Principles for Success*, Springer 2020
4. Salazar M. Lant T. Facilitating Innovation in Interdisciplinary Teams: The Role of Leaders and Integrative Communication, In: Lotrecchiano G. Misra S eds. *Transdisciplinary Communication in Team*s, Informing Science Institute 2019.
5. Clark, LA Watson, D. Constructing validity: Basic issues in objective scale development. In: Kazdin AE ed. *Methodological issues and strategies in clinical research* 4th ed.,). American Psychological Association 2016: 187–203  [https://doi.org/10.1037/14805-012](https://psycnet.apa.org/doi/10.1037/14805-012)
6. Hitlin P. Turkers in this canvassing: young, well-educated and frequent users. *Pew Res Cen* 2016: *437*.
7. Sudman, S., Bradburn, NM., Schwarz, N. *Thinking about answers: The application of cognitive processes to survey methodology*. Jossey-Bass. 1996
8. Worthington, RL Whittaker, TA. Scale development research: A content analysis and recommendations for best practices. C*ouns psychol*, 2006. 34(6), 806-838.
9. Dillman, DA., Smyth, JD., & Christian, LM. *Internet, phone, mail, and mixed-mode surveys: The tailored design method.* John Wiley & Sons. 2014.
10. Tabachnick, B.G Fidell, LS & Ullman, JB. *Using multivariate statistics* Pearson 2013 6: 497-516.
11. Fabrigar LR Wegener DT MacCallum RC Strahan EJ. Evaluating the use of exploratory factor analysis in psychological research. *Psych meth*, 1999 4(3), 272.
12. Gorsuch, RL. Common factor analysis versus component analysis: Some well- and little-known facts. *Multi behav res*, 1990 25(1), 33-39.
13. Faraj S, Sproull L. Coordinating expertise in software development teams. *Manage Sci,* 2000; *46*(12), 1554-1568.<http://www.jstor.org/stable/2661533>
14. Knight, D. Pearce, CL Smith, et al. Top Management Team Diversity, Group Process, and Strategic Consensus, *Strat Manag J*, 1999;20: 445-465
15. Connelly, CE., Zweig, D., Webster, J., et al. Knowledge hiding in organizations. *J Organ Beh*, 2012.;*33*(1), 64-88. https://www.jstor.org/stable/pdf/41415737.pdf
16. Morizot, J. Construct validity of adolescents’ self-reported big five personality traits: Importance of conceptual breadth and initial validation of a short measure. *Assess,* 2014.;*21*(5), 580–606.
17. Wang, XH, Howell, JM. Exploring the dual-level effects of transformational leadership on followers. *J App Psychol, 2010 95*(6), 1134-1144
18. Pearce, CL Sims HP Jr. Vertical versus shared leadership as predictors of the effectiveness of change management teams: An examination of aversive, directive, transactional, transformational, and empowering leader behaviors. *Gr Dyn: The Res Pract, 2002 6*(2) 172–197.
19. Jarvenpaa, SL, Knoll K Leidner DE. Is Anybody out There? Antecedents of Trust in Global Virtual Teams. *J Manag Info Syst*, 1998 *14*(4), 29–64.
20. Tierney P. Farmer, SM. [Creative Self-Efficacy: Its Potential Antecedents and Relationship to Creative Performance](https://journals.aom.org/doi/full/10.5465/3069429) 2002 *Acad Manag J* [45 (6](45%20(6)**)** <https://doi.org/10.5465/3069429>
21. Lewis, K. Measuring transactive memory systems in the field: Scale development and validation. *J Appl Psych* 2003 587-604 88(4)
